# Elevated Familial Cardiovascular Burden Among Adolescents With Familial Bipolar Disorder

**DOI:** 10.3389/fpsyt.2019.00008

**Published:** 2019-01-29

**Authors:** Simina Toma, Lisa Fiksenbaum, Danielle Omrin, Benjamin I. Goldstein

**Affiliations:** ^1^Centre for Youth Bipolar Disorder, Sunnybrook Health Sciences Centre, Toronto, ON, Canada; ^2^Department of Psychiatry, University of Toronto, Toronto, ON, Canada; ^3^Department of Pharmacology, University of Toronto, Toronto, ON, Canada

**Keywords:** bipolar, metabolic, family history, cardiovascular, adolescents

## Abstract

**Background:** Bipolar disorder (BD) is one of the most heritable medical conditions, and certain phenotypic characteristics are especially familial in BD. BD is also strongly associated with elevated and premature cardiovascular disease (CVD) morbidity and mortality. Thus, far, little is known regarding the familiality of cardiovascular risk in BD. We therefore examined the extent of CVD-related conditions among relatives of: adolescents with BD with a family history of BD (familial BD), adolescents with BD without a family history of BD (non-familial BD) and healthy controls (HC).

**Materials and Methods:** The sample included 372 adolescents; 75 with familial BD, 96 with non-familial BD, and 201 HC. Parents of the adolescents completed the CARDIA Family Medical History interview regarding the adolescents' first- and second- degree adult relatives. We computed a “cardiovascular risk score” (CRS) for each relative, based on the sum of the presence of diabetes, hypertension, obesity, dyslipidemia, stroke, angina, and myocardial infarction (range 0–7). Primary analyses examined for group differences in mean overall CRS scores among first and second- degree relatives combined, controlling for age, sex, and race. Secondary analyses examined first- and second-degree relatives separately, controlling for age, sex, and race.

**Results:** There were significant between-group differences in CRS in first- and second- degree relatives combined, following the hypothesized ordering: CRS was highest among adolescents with familial BD (1.14 ± 0.78), intermediate among adolescents with non-familial BD (0.92 ± 0.79) and lowest in HC (0.76 ± 0.79; *F* = 6.23, *df* = 2, *p* = 0.002, η${}_{\text{p}}^{2}$ = 0.03). There was a significant pairwise difference between adolescents with familial BD and HC (*p* = 0.002, Cohen's *d* = 0.49). A similar pattern of between-group differences was identified when first-degree and second-degree relatives were examined separately.

**Limitations:** familial cardiovascular burden was determined based on parent interview, not evaluated directly.

**Conclusions:** Adolescents with BD with a family history of BD have elevated rates of CVD-related conditions among their relatives. This may be related to genetic overlap between BD and CVD-related conditions, shared environmental factors that contribute to both BD and CVD-related conditions, or a combination of these factors. More research is warranted to better understand the interaction between familial risk for BD and CVD, and to address this risk using family-wide preventive approaches.

## Introduction

Bipolar Disorder (BD) is a chronic mood disorder with a strong genetic contribution (1). Studies have estimated that heritability of BD is 0.8, although the exact pattern of heritability and implicated genes are not yet elucidated (2). In addition to an 8–20 fold increase in risk of developing BD, relatives of those with BD are at higher risk for other psychiatric conditions such as depressive disorders, anxiety disorders, attention deficit hyperactivity disorder (ADHD), and substance use disorders (3, 4). Studies have also reported neurocognitive differences between unaffected relatives of BD probands and controls, potentially related in part to obesity in the unaffected relatives (5–7). Furthermore, in those with BD with a family history of BD (familial BD), several characteristics, and markers of severity such as substance use disorders, psychosis, suicidality, and level of social functioning may be shared by BD probands from the same family, suggesting that certain phenotypes may congregate in familial BD (8).

There is a known link between BD and cardiovascular disease (CVD) risk, with excessive and premature morbidity and mortality replicated in samples from various countries (9–15).^.^CVD and its complications are the leading cause of mortality in BD, and the most common medical conditions in BD (16, 17). In comparison to healthy controls (HC) individuals with BD have an adjusted CVD mortality rate ratio of 1.5–2.5 and CVD mortality 10 years earlier than in the general population (16, 18). In addition, the age of onset of new CVD was found to be up to 17 years premature in BD (19). Metabolic syndrome and its components of obesity, hypertension, high cholesterol levels and type II diabetes are also elevated in those with BD (20–22). This association between CVD and BD is in excess of what can be explained by psychotropic medication, lifestyle behaviors and even traditional CVD risk factors (11, 19, 23–25).

There is a paucity of studies on the topic of cardiovascular burden in unaffected relatives of BD probands. One study found lower plasma high-density lipoprotein (HDL) cholesterol and increased omega-6 fatty acids in adult unaffected first-degree relatives of adults with BD in comparison to controls (26). A second study found increased prevalence of cardiovascular-related conditions (diabetes, hypertension, hyperlipidemia, and coronary artery disease) with a risk rate ratio of 4.8 in affected and unaffected adult first-degree relatives of probands with schizophrenia, schizoaffective disorder, bipolar subtype, and BD with psychotic symptoms in comparison to controls (27). Adult offspring of BD subjects were included. There was no effect of the specific psychiatric diagnosis of the proband on cardiovascular risk in the relative (27). A third study examining first-, second- and third-degree relatives of subjects with BD within a large extended family did not find differences in the rate of metabolic syndrome or obesity rates in BD relatives vs. HC, but found higher total cholesterol, LDL and triglycerides, lower HDL, and abnormal glucose in BD relatives (28). An analysis of children and adolescents with a second- or third-degree family history of BD (mean age 11.6 years old) in contrast to HC (mean age 7.8 years old), controlling for age, found higher rates of elevated LDL in the BD relatives than in HC, although there were higher rates of elevated triglycerides and low HDL in the HC group (28).

Importantly, there is a strong familial aggregation of metabolic syndrome and its components in non-psychiatric samples (29–31). A large study in psychiatrically healthy young adults found anomalous blood pressure (BP), cholesterol and glucose profiles in those with parental history of CVD-related conditions including myocardial infarction, stroke, diabetes, hypertension and obesity (32). In the only study on the topic, a family history of type II diabetes was associated with metabolic abnormalities such as insulin resistance, fasting blood glucose, higher body mass index (BMI), and waist circumference in adult women with BD, and the impact of a family history of type II diabetes was greater in those with BD than in controls (33).

Given the paucity of research on this important topic, particularly in relation to early-onset BD, we compared the cardiovascular burden among adolescents, and hypothesized that cardiovascular burden would be highest among adolescents with familial BD, followed by adolescents with non-familial BD, followed by HC adolescents.

## Materials and Methods

This study included 372 adolescent participants (171 BD, 201 HC) between the ages of 13–20 years old. Adolescents with BD-I, -II, or -Not Otherwise Specified (NOS) were recruited from a tertiary subspecialty clinic in an academic health sciences center in Toronto, Canada, and HC were recruited from the community via advertisements in the Greater Toronto Area. All participants were English-speaking. HC had no lifetime history of mood or psychotic disorders, or substance use disorders within the preceding 3 months. In addition, HC did not have a first- or second-degree family history of BD or psychotic disorders. HC were also excluded if they had a history of cardiac, autoimmune or inflammatory illness, neurological or cognitive impairment or were treated with anti-inflammatory, anti-platelet, anti-lipidemic, anti-hypertensive, or hypoglycemic agents including insulin and metformin.

Adolescent psychiatric diagnoses were made using the Schedule for Affective Disorders and Schizophrenia for School Age Children, Present, and Life Version (K-SADS-PL) (34), a semi-structured interview completed with adolescents and parents to ascertain current and lifetime history of psychiatric disorders. The KSADS Depression Rating Scale (DRS) (35) and the KSADS Mania Rating Scale (MRS) (36) were used in place of the mood section in the K-SADS-PL. Diagnoses were confirmed by a child-adolescent psychiatrist. BD-NOS was defined using criteria previously operationalized by the Course and Outcome of Bipolar Illness in Youth (COBY) study group (37): Elevated and/or irritable mood, plus (1) two *Diagnostic and Statistical Manual of Mental Disorders*, 4th ed. (DSM-IV) (38) manic symptoms (3 if only irritable mood is reported), (2) change in functioning, (3) mood, and symptom duration of at least 4 h during a 24 h period, and (4) at least four cumulative 24 h periods of episodes over the participants' lifetime that meet the mood, symptom severity, and functional change criteria. Overall, the participants' general level of functioning was evaluated using the Children's Global Assessment Scale (CGAS) (39), which was administered as an interview. Socio-economic Status (SES) was evaluated using the Hollingshead Four Factor Index (40).

Family psychiatric history in all first- and second-degree relatives was evaluated using the Family History Screen interview (41). The Coronary Artery Risk Development in Young Adults Study (CARDIA) Family Medical History was completed as an interview with adolescents and their parents, regarding adolescents' first- and second-degree adult relatives (42). Adolescents and a parent were interviewed and provided information on second-degree relatives of the adolescent, including aunts, uncles and grand-parents. These second-degree relatives were not directly interviewed, nor were their medical records accessed. The current study focused on family history of diabetes, hypertension, obesity, dyslipidemia, stroke, angina, and myocardial infarction. A “Cardiovascular Risk Score” (CRS) was computed for each relative based on the sum of the number of these conditions present (score of 0–7). Given the young age of participants' siblings, first-degree relatives included only parents. Familial mean CRS scores were calculated for parents and for combined first- and second-degree relatives.

All participants, as well as one parent or guardian, provided written informed consent prior to study participation. The study was approved by the local research ethics board.

### Anthropomorphic Variables

Measures of height and weight were available for 339 adolescents, and systolic blood pressure (SBP) and diastolic blood pressure (DBP) were available for 344 adolescents. Body mass index (BMI) was computed by dividing weight in kilograms (kg) by height in meter squared (m^2^) as previously described (43). Percentiles were determined using the BMI-for-age percentile based on Centers for Disease Control (CDC) growth charts, applicable for youth under the age of 20 (44).

### Statistical Analysis

Analyses were performed using SPSS, version 24 (IBM Corp., Armonk, N.Y., USA). Participants were divided into three groups: BD adolescents with family history of BD (familial BD), BD adolescents without family history of BD (non-familial BD), and HC adolescents. Group differences were evaluated using one-way ANOVA for dimensional measures and chi-square tests for dichotomous measures. To test our primary hypothesis, a one-way ANCOVA (controlling for age, sex, and race) was used to compare CRS across the three groups. Omnibus tests comparing CRS across the groups were followed by *post-hoc* pairwise comparisons of CRS.

## Results

### Demographic and Clinical Characteristics

Table 1 presents demographic and clinical variables for all study participants; descriptive statistics are presented for BD participants in Table 2. The sample included 372 adolescents: 75 with familial BD, 96 with non-familial BD, and 201 HC. 372 parents were interviewed (one for each adolescent participant) and provided information regarding their own medical history along with that of co-parents and second-degree relatives. In total, information regarding medical history was obtained regarding 2,797 second degree relatives, among which 561 were relatives of adolescents with familial BD, 691 were relatives of adolescents with non-familial BD, and 1,545 were relatives of healthy adolescents. There were significant differences between the adolescent groups in terms of age, sex, and race. A total of 19.9% of the HC group had at least one lifetime psychiatric diagnosis, including anxiety disorders (8.5%), ADHD (11.1%), obsessive compulsive disorder (OCD; 1%), and oppositional defiant disorder (ODD; 1%). Furthermore, 1% of HC had a lifetime history of antidepressant use, and 4% had a lifetime history of stimulant use.

**Table 1:** Demographic and clinical variables among 372 adolescents.

	**Participants**			**Statistics**		
	**Familial BD (*n* = 75)**	**Non-familial BD (*n* = 96)**	**HC (*n* = 201)**	***F*/ χ^2^**	**Cramer's V/ η*p*^**2**^**	***P*-value**
Age, years (±SD)	16.5 ± 1.46	16.8 ± 1.50	16 ± 1.82	7.68	0.04	< 0.001
Females (%)	48 (64%)	66 (68.8%)	104 (51.7%)	8.88	0.15	0.012
Race (%Caucasian)	63 (84%)	75 (78.1%)	112 (55.7%)	26.82	0.27	< 0.001
SES (±SD)	49.19 ± 12.08	48.95 ± 14.18	52.36 ± 11.13	3.41	0.02	0.034

*BD, Bipolar Disorder; HC, Healthy Controls; SES, Socio-economic Status; SD, Standard Deviation*.

**Table 2:** Clinical characteristics among 171 adolescents with bipolar disorder.

Age at onset (±SD)	14.66 ± 2.73
BD subtype (%)	
BD-I	45 (26.3%)
BD-II	65 (38%)
BD-NOS	61 (35.7%)
Lifetime comorbidity (%)	
Substance use disorder	59 (35.5%)
ADHD	79 (47.6%)
Anxiety disorder	132 (79.5%)
OCD	29 (17.5%)
ODD	54 (32.5%)
Lifetime medication use (%)	
SGA	92 (55.4%)
Lithium	28 (16.9%)
Antimanic/Anticonvulsant	13 (7.8%)
Antidepressant (SSRI)	62 (37.3%)
Stimulants	37 (22.3%)
Clinical scores (±SD)	
Mania score-current	18.43 ± 12.44
Mania score-lifetime most severe	29.60 ± 9.05
Depression score-current	19.86 ± 12.84
Depression score-lifetime most severe	31.59 ± 10.21
CGAS—Current episode	52.99 ± 10.57
CGAS—Highest past	60.21 ± 11.58
CGAS—Lowest past	42.29 ± 8.73

*ADHD, Attention Deficit/Hyperactivity Disorder; BD, Bipolar Disorder; CGAS, Children's Global Assessment Scale; OCD, Obsessive Compulsive Disorder; ODD, Oppositional Defiant Disorder; SD, Standard Deviation; SGA, Second Generation Antipsychotics; SSRI, Selective Serotonin Reuptake Inhibitor*.

Anthropomorphic variables are presented in Table 3. There were significant between-group differences in BMI percentile (*F* = 4.40, *p* = 0.01, η${}_{\text{p}}^{2}$ = 0.03). When controlling for age, sex and race, average systolic, and average diastolic BP were also significantly higher in the BD groups in comparison to HC (respectively *F* = 4.85, *p* = 0.008, η${}_{\text{p}}^{2}$ = 0.03; *F* = 6.86, *p* < 0.001, η${}_{\text{p}}^{2}$ = 0.04).

**Table 3:** Anthropomorphic variables among adolescents.

	**Participants**			**Statistics**		
	**Familial BD**	**Non-familial BD**	**HC**	***F*/χ^2^**	**Cramer's V/ η*p*^**2**^**	***P*-value**
BMI percentile (*n* = 336)	62.67 ± 27.67 (*n* = 65)	66.07 ± 26.08 (*n* = 77)	56.01 ± 26.88 (*n* = 194)	4.4	0.03	0.013
Systolic blood pressure (*n* = 344)	115.70 ± 19.07 (*n* = 67)	114.72 ± 16.44 (*n* = 79)	110.04 ± 13.63 (*n* = 198)	4.85	0.03	0.008
Diastolic blood pressure (*n* = 344)	72.75 ± 9.40 (*n* = 67)	70.87 ± 11.01 (*n* = 79)	67.5 ± 7.68 (*n* = 198)	6.86	0.04	0.001

*BD, Bipolar Disorder; HC, Healthy Controls; SES, Socio-economic Status; SD, Standard Deviation*.

### Cardiovascular Risk Score (CRS)

Overall familial CRS (i.e., first and second-degree relatives combined) differed significantly across groups in the hypothesized direction: highest among familial BD (1.14 ± 0.78), intermediate among non-familial BD (0.92 ± 0.79), and lowest among HC (0.76 ± 0.79) (see Table 2) (*F* = 6.23, *p* = 0.002, η${}_{\text{p}}^{2}$ = 0.03). Planned pair-wise comparisons indicated a significant difference between the familial BD and HC groups (*p* = 0.002, Cohen's *d* = 0.49), and non-significant differences between the familial BD and non-familial BD relatives (*p* = 0.19; Cohen's *d* = 0.28) as well as between the non-familial BD and HC relatives (*p* = 0.34; Cohen's *d* = 0.20).

CRS among only first-degree relatives (i.e., parents) followed the same pattern: highest among familial BD (0.65 ± 0.60), intermediate among non-familial BD (0.48 ± 0.60), and lowest among HC (0.32 ± 0.61) (see Table 2) (*F* = 8.63, *p* < 0.001, η${}_{\text{p}}^{2}$ = 0.05). Planned pair-wise comparisons indicated a significant difference between familial BD and HC (*p* < 0.001; Cohen's *d* = 0.56) but not between familial BD and non-familial BD (*p* = 0.11; Cohen's *d* = 0.30) or between non-familial BD and HC (*p* = 0.15; Cohen's *d* = 0.26).

Finally, CRS among only second-degree relatives also followed the same pattern: highest among familial BD (0.67 ± 0.47), intermediate among non-familial BD (0.57 ± 0.47), and lowest among HC (0.49 ± 0.48) (see Table 2) (*F* = 3.96, *p* = 0.02, η${}_{\text{p}}^{2}$ = 0.02). Planned pair-wise comparisons indicated a significant difference between familial BD and HC (*p* = 0.02, Cohen's *d* = 0. 38) but not between familial BD and non-familial BD (*p* = 0.45, Cohen's *d* = 0.21) or between non-familial BD and HC (*p* = 0.58, Cohen's *d* = 0.17).

## Discussion

This study found that cardiovascular risk, based on a score defined by the combination of diabetes, hypertension, obesity, dyslipidemia, stroke, angina, and myocardial infarction, was highest in relatives of adolescents with familial BD, followed by relatives of adolescents with non-familial BD, followed by relatives of HC adolescents. Whereas, familial CRS differed significantly between adolescents with familial BD and HC, familial CRS among adolescents with non-BD did not differ significantly from the other groups. A similar pattern, including the ordering effect and between-group effect sizes, was found for combined first- and second-degree relatives, and for first- and second- degree relatives examined separately.

In addition to the known link between BD and cardiovascular risk, the limited number of prior studies on the topic of cardiovascular burden in adult BD relatives also found elevated rates of dyslipidemia and insulin resistance, including those unaffected by BD, in comparison to HC relatives (20, 26–28). The current study extends prior findings by showing that relatives of adolescents with BD have particularly elevated cardiovascular risk in the context of a family loading of BD, which has not been previously described. Relatives of adolescents with non-familial BD were intermediate between the other groups. This ordering effect could reflect differential loading of genetic and/or environmental risk for CVD-related conditions. Our findings could be due to familial BD being a more severe phenotype, shared genetic factors or biological processes such as inflammation, familial psychiatric burden, or environmental influences such as patterns of exercise and substance use (45–48). However, the current study was not designed to evaluate these hypotheses.

Family history of BD or other mood disorders has been associated with an earlier age of onset of BD, higher rates of psychiatric comorbidities and an overall more severe course of illness among people with BD (8, 45, 49, 50). More severe course of BD, in turn, is associated with increased risk of CVD and CVD mortality (19, 51–53). Relatedly, prior cross-sectional studies found that CVD risk factors, including metabolic syndrome and its components, are associated with increased functional impairment, suicide attempts and symptom severity in BD (54–56). Taking together prior findings and current findings, it appears that there is an interweaving of psychiatric and cardiovascular burden in BD, and that this interweaving is familial.

Prior studies provide context for the potential genetic contributions to current findings. Independently, BD and metabolic syndrome are each known to have a strong familial aggregation, yet little is known about their interaction (1, 29–31). A study found that susceptibility gene TCF7L2 conferred an increased risk of BD in the presence of elevated BMI, suggesting an interaction between an interaction between obesity and BD risk (57). Furthermore, genetic variants thought to be implicated in BD such as BDNF, MTHFR, GNAS, and CACNA1C/D, have been hypothesized to overlap between BD and CVD, conferring risk of mood disorders in addition to risk of hypertension, type 2 diabetes, obesity, and dyslipidemia (46, 58). Dysregulation of the inflammatory system with increased pro-inflammatory markers such as cytokines, especially during acute mood episodes has been well-described in BD, and may comprise a familial trait in BD pedigrees (59–63). It is well-recognized that immune dysfunction and chronic inflammation are associated with CVD and related risk factors (64–66). It has been proposed that a genetic predisposition to inflammation could be linked to both BD and CVD-related conditions (67).

Similar to putative genetic contributions, prior studies also provide context for the potential environmental contributions to current findings. For example, obesity and metabolic syndrome have been associated with pregnancy complications such as gestational hypertension or preeclampsia, as well as future risk of obesity and heart disease in the offspring (68, 69). Maternal cardiovascular risk factors during pregnancy have been linked with an increased risk of ADHD, autism spectrum disorder, eating disorders, and psychosis in offspring (70, 71). Although studies have yet to link maternal gestational cardiovascular risk factors with risk of BD in offspring, it is known that these risk factors are increased among pregnant women with BD, which one can speculate is also relevant to the transmission of BD to the offspring, and to the cross-risk of CVD-related conditions and BD (72).

Another environmental factor that may underlie our findings is lifestyle. For example, an individual's physical activity is associated with physical activity among relatives (73, 74) and some studies have found that adults with BD tend to be less physically active than the general population (75–79). Similarly, there is also evidence that adolescents with BD are less likely to engage in moderate-vigorous physical activity than controls (80). Sedentary lifestyle has been associated in the general population with metabolic syndrome and CVD morbidity and mortality (47, 81). While there are genetic factors that contribute to physical activity, reduced physical activity in relatives also comprises an environmental factor that influences behavior (82, 83). Similar considerations apply to other CVD risk factors such as cigarette smoking (84–87). Furthermore, adverse childhood experiences such as poverty, family conflicts, maltreatment, neglect, or peer victimization have been linked with both cardiovascular burden and psychopathology, and this association is thought to be mediated by both psychological and neurobiological factors (11, 88, 89) Indeed, there is a hypothesized synergistic interaction between genetic predisposition, epigenetic factors such as DNA methylation in the presence of early adversity, health behaviors and subsequent risk for mood disorders and CVD (88, 90–92).

## Limitations

Several limitations may have impacted our findings. First, the data collected from adolescents and their parents is indirect and based on their knowledge of family history. The absence of direct assessment of the relatives' cardiovascular health or access of their medical records is a major limitation of this study. Future studies would benefit from directly examining medical records and directly evaluating for psychiatric disorders and directly measuring cardiovascular risk factors. Given known disparities in the recognition and treatment of CVD-related conditions among people with BD and other severe psychiatric conditions, our findings may be biased toward lower CRS scores. Second, although we have controlled for key demographic variables, the study methods and sample size do not allow us to address questions regarding the effect of BD independent of variables such as psychiatric comorbidities and lifestyle. Nonetheless, prior epidemiologic studies in predominantly untreated samples have verified that the BD-CVD link is independent of these important considerations (19, 25). Finally, the CRS was computed as a sum score of conditions that are inter-related, and includes cardiovascular risk factors (e.g., hypertension) alongside fully manifest vascular disorder (e.g., myocardial infarction). Larger samples would enable alternative proxies for cardiovascular burden, and would allow for evaluation of fully manifest vascular disorder while controlling for cardiovascular risk factors (as has been done in studies evaluating cardiovascular risk among those with BD). Counter-balancing these limitations is the importance of gaining insights regarding the BD-CVD link; to this end, the current study is the first on the topic that is focused on adolescents, and the first study in any age group to evaluate the link between familiality of BD and familiality of cardiovascular risk.

## Future directions

In addition to the aforementioned future directions within the limitations section, studies are warranted that examine for correlation between probands and their relatives regarding cardiovascular burden, and that evaluate whether these correlations differ across groups (i.e., familial BD, non-familial BD, HC). Because of the very large samples required to evaluate low-frequency “hard” endpoints, such as myocardial infarction and stroke, administrative database studies based on large population samples would provide complementary information to that available in clinical cohort studies. As with any observational study, prospective design would offer advantages with regard to causal inferences and mechanisms. Studies that include genetic markers and biomarkers beyond glucose and lipids (e.g., inflammatory markers, neurotrophic factors, oxidative stress markers) would enable further evaluation of potential bridges linking BD and CVD-related conditions. Finally, it will be important to move toward modifying assessment and treatment approaches that are informed by the BD-CVD link. For example, treatment approaches for those with BD may benefit from the assessment of medical and psychiatric family history and identification of those at higher risk. In addition, future studies evaluating behavioral and pharmacological approaches to prevention and treatment of CVD-related conditions in BD could benefit from incorporating familial considerations.

## Conclusions

In conclusion, we found that adolescents with BD with a family history of BD have elevated rates of CVD-related conditions among their relatives. This may be related to genetic overlap between BD and CVD-related conditions, shared environmental factors that contribute to both BD and CVD-related conditions, or a combination of these factors. More research is warranted to better understand the interaction between familial risk for BD and CVD. The possible interaction between BD familial loading and CVD loading opens the opportunity to integrate familial medical and psychiatric history during assessment, and opens the opportunity to use this information to inform prevention and treatment strategies. Involvement of family members may be beneficial due to the shared environmental factors and familial nature of BD and cardiovascular risk.

## Ethics Statement

Sunnybrook Health Sciences Center Research Ethics Board Written consent.

## Author Contributions

LF and ST performed the analyses. ST primarily wrote the manuscript. All authors participated in iterative revisions of the manuscript and participated in the conception and design of the analysis.

### Conflict of Interest Statement

The authors declare that the research was conducted in the absence of any commercial or financial relationships that could be construed as a potential conflict of interest.
